# Spicing up endogenous neural stem cells: aromatic-turmerone offers new possibilities for tackling neurodegeneration

**DOI:** 10.1186/scrt517

**Published:** 2014-11-17

**Authors:** Steven W Poser, Andreas Androutsellis-Theotokis

**Affiliations:** Department of Medicine, Technische Universität Dresden, Fetscherstrasse 74, Dresden, 01307 Germany

## Abstract

There is a growing interest in the therapeutic utility of compounds derived from *Curcuma longa*, an herb of the Zingiberaceae family that has been part of traditional medicine for centuries. Recent reports suggest that bioactive compounds isolated from the rhizome of these plants can address two key aspects of brain injury following stroke that must be dealt with for functional recovery to occur: the moderation of neuroinflammation, and the mobilization of endogenous stem cells resident in the nervous system. Defining their mechanism of action remains a question, but emerging evidence may point towards one shared with more classic modulators of neural stem cell proliferation and survival.

Despite spending tremendous amounts of effort and money, there remain few treatment options available for those who have suffered a brain injury as the consequence of a stroke. Reparative mechanisms resident in the brain are often insufficient to address the damage caused by such an insult, and current therapeutic approaches, whether they are cell based, compound based, or a combination of the two, are too often inadequate to produce long-lasting benefits to the patient. It is a daunting task to identify new avenues of treatment, especially given the many different challenges that need to be overcome to facilitate repair of the nervous system. The paper by Hucklenbroich and colleagues presented in a previous issue of *Stem Cell Research and Therapy*, provides evidence that aromatic-turmerone, an extract from the herb *Curcuma longa,* could provide one of those avenues [1].

One aspect to address is providing trophic support to the damaged tissue, giving cells the opportunity to recover and return to normal function. Neural stem cells, activated as a consequence of injury, play a significant role in the regenerative process. This is primarily through their production of neurotrophic factors and cytokines such as brain-derived neurotrophic factor and Sonic hedgehog [2, 3], instead of harnessing their ability to differentiate and physically replace damaged neurons, astrocytes, or oligodendrocytes. A second issue to address is the excessive neuroinflammation associated with neurological damage. While initial inflammatory events provide important signals for attracting immune cells to deal with the acute damage as well as recruit neural stem cells [4], chronic inflammatory mediators produced through activated microglia oppose the recovery of damaged tissue through production of factors such as chemokines and free radicals [5, 6]. Therefore, a valuable approach would be one that is multimodal in its cellular action, appropriately modulating the inflammatory process while activating endogenous neural stem cells, allowing for the establishment of a regenerative microenvironment that facilitates innate repair and functional recovery.

Traditional herbal medicines are becoming increasingly focused upon as the search for effective therapies continues. Some in particular are based upon extracts from a member of the Zingiberaceae family, *Curcuma longa*. It has long been used as part of traditional medicine for treating a broad spectrum of indications. There are a growing number of investigations into the bioactive compounds it contains, with curcumin garnering most of the attention thus far, having multiple published reports demonstrating its effects on the survival of various cancer types as well as treating microbial infection, among other applications [7]. More recently, another component, aromatic-turmerone, has also moved into the spotlight as a potential therapy with appealing qualities for treating neuronal damage. It has documented anti-inflammatory properties, in particular towards the activity of microglia associated with neuroinflammation, meaning it satisfies the first criterion for that sought-after therapeutic approach. What about the second criterion, the ability to activate endogenous neural stem cells? That piece of the puzzle is provided by a manuscript from Hucklenbroich and colleagues [1] describing the effects of aromatic-turmerone on fundamental neural stem cell properties. They demonstrate the interesting finding that, while treatment of fetal neural stem cells *in vitro* during expansion yields increased proliferation, treatment during differentiation promotes an increase in the number of generated neurons. They utilize positron emission tomography imaging to show the effects of intracerebroventricular injection of aromatic-turmerone on the mobilization of neural stem cells from both the subventricular zone and hippocampus. It represents an important step in defining the beneficial cellular mechanism this class of compounds can exert on the regenerative process in the nervous system and opens an avenue for their therapeutic use. The fact that independent studies utilizing curcumin were published almost simultaneously with the findings of Hucklenbroich and colleagues [8, 9] showcases the therapeutic potential of this family of plants.

Identifying the mechanism of action for aromatic-turmerone will be key for it to move forward as a useful therapeutic. In the case of its anti-inflammatory effects, the details are coming to light, as it inhibits NF-κB, JNK, and p38 activity in microglia [10]. However, how it modulates the proliferation and differentiation of neural stem cells is not understood. One possibility is that it acts upon the STAT3-Ser/Hes3 signaling axis, an emerging regulator of neural stem cell growth and survival [11]. It is a distinct branch of the Notch signaling pathway activated by fibroblast growth factor 2, Delta4, angiopoietin 2, and insulin, all of which are able to increase neural stem cell number. Treatment with these factors, especially in combination, results in the mobilization of endogenous neural stem cells and has powerful effects in recovery models of ischemic stroke and Parkinson’s disease [12, 13]. The activity of the JAK and p38MAP kinase oppose this pathway. p38 suppresses STAT3 phosphorylation on the serine 727 residue, a key component of the pathway. If aromatic-turmerone inhibits p38 activity in neural stem cells as it does in microglia, it would inactivate an important negative feedback loop into the pathway, allowing for higher and more sustained activity in response to mitogenic signals.

Another possible mechanism of aromatic-turmerone action worth considering is that, like curcumin, it may suppress STAT3 tyrosine 705 phosphorylation. In the context of cancer, curcumin treatment results in a reduction in STAT3 tyrosine 705 phosphorylation levels [8, 14–16]. If aromatic-turmerone does the same in neural stem cells, it would operate as a JAK inhibitor does, opposing the differentiation of neural stem cells towards a glial fate and allowing STAT3 serine 727 phosphorylation to dominate, a state that results in increased STAT3-Ser/Hes3 signaling axis activity and enhanced cell number. Straightforward experiments could be performed to quickly assess the involvement of this mechanism of action. This would provide an important step forward in establishing therapeutic paradigms incorporating aromatic-turmerone for providing benefit to those suffering from debilitating neurological conditions.
